# Silver nanoparticles reduce brain inflammation and related neurotoxicity through induction of H_2_S-synthesizing enzymes

**DOI:** 10.1038/srep42871

**Published:** 2017-03-02

**Authors:** Daniel A. Gonzalez-Carter, Bey Fen Leo, Pakatip Ruenraroengsak, Shu Chen, Angela E. Goode, Ioannis G. Theodorou, Kian Fan Chung, Raffaella Carzaniga, Milo S. P. Shaffer, David T. Dexter, Mary P. Ryan, Alexandra E. Porter

**Affiliations:** 1 Department of Materials and London Centre for Nanotechnology, Imperial College London, Exhibition Road, London, SW7 2AZ, UK; 2 Parkinson’s Disease Research Unit, Centre for Neuroinflammation and Neurodegeneration, Division of Brain Sciences, Imperial College London, Hammersmith Hospital Campus, London, W12 0NN, UK; 3 Central Unit for Advanced Research Imaging, Faculty of Medicine, University of Malaya, 50603 Kuala Lumpur, Malaysia; 4 National Heart and Lung Institute, Imperial College London, Guy Scadding Building, Cale Street, London, SW3 6LY, UK; 5 Electron Microscopy Science Technology Platform, The Francis Crick Institute, Lincoln’s Inn Fields Laboratory, 44 Lincoln’s Inn Fields, London, WC2A 3LY, UK; 6 Department of Chemistry and London Centre for Nanotechnology, Imperial College London, Exhibition Road, London, SW7 2AZ, UK

## Abstract

Silver nanoparticles (AgNP) are known to penetrate into the brain and cause neuronal death. However, there is a paucity in studies examining the effect of AgNP on the resident immune cells of the brain, microglia. Given microglia are implicated in neurodegenerative disorders such as Parkinson’s disease (PD), it is important to examine how AgNPs affect microglial inflammation to fully assess AgNP neurotoxicity. In addition, understanding AgNP processing by microglia will allow better prediction of their long term bioreactivity. In the present study, the *in vitro* uptake and intracellular transformation of citrate-capped AgNPs by microglia, as well as their effects on microglial inflammation and related neurotoxicity were examined. Analytical microscopy demonstrated internalization and dissolution of AgNPs within microglia and formation of non-reactive silver sulphide (Ag_2_S) on the surface of AgNPs. Furthermore, AgNP-treatment up-regulated microglial expression of the hydrogen sulphide (H_2_S)-synthesizing enzyme cystathionine-γ-lyase (CSE). In addition, AgNPs showed significant anti-inflammatory effects, reducing lipopolysaccharide (LPS)-stimulated ROS, nitric oxide and TNFα production, which translated into reduced microglial toxicity towards dopaminergic neurons. Hence, the present results indicate that intracellular Ag_2_S formation, resulting from CSE-mediated H_2_S production in microglia, sequesters Ag^+^ ions released from AgNPs, significantly limiting their toxicity, concomitantly reducing microglial inflammation and related neurotoxicity.

The widespread use of silver nanoparticles (AgNPs) in various consumer products, ranging from food packaging to antibacterial sprays and water purifiers^1^,^2^, has raised concerns regarding their potential adverse effects on human health. Indeed, AgNPs are able to induce cytotoxicity in human lung, skin and fibroblast cells^3^,^4^,^5^. In relation to the central nervous system (CNS), AgNPs have been shown to be able to cross the blood-brain barrier^6^,^7^ and accumulate in the brain following ingestion^8^ and inhalation^9^,^10^. Furthermore, a growing body of evidence indicates AgNPs are able to directly induce cytotoxicity in neurons *in vitro*^11^,^12^,^13^,^14^,^15^, and cause neurodegeneration *in vivo* following oral^16^,^17^, gastric^18^ or nasal administration^19^. However, the precise mechanisms of neurodegeneration are not fully understood. Therefore, the effects of AgNPs on other CNS cell types and their contribution to AgNP-induced neurodegeneration need to be more thoroughly examined.

Microglia are the brain’s resident immune cells, responsible for mounting protective inflammatory reactions to destroy invading pathogens^20^. However, excessive microglial inflammation is capable of inducing collateral neuronal damage through over-production of pro-inflammatory factors such as the pro-apoptotic protein tumour necrosis factor (TNF)-α, reactive oxygen species (ROS) and nitric oxide (NO), and is implicated in the chronic neuronal death seen in neurodegenerative diseases such as Parkinson’s disease (PD) and Alzheimer’s disease^21^,^22^,^23^. Furthermore, nanomaterials have been shown to be able to induce brain inflammation and changes related to neurodegenerative diseases^24^,^25^,^26^,^27^. Therefore, it is important to examine the effect of AgNPs on microglial cell viability and inflammation to fully understand AgNP-induced neurodegeneration and whether AgNP-exposed microglia exacerbate this process.

In addition to mounting inflammatory reactions for pathogen destruction, microglia are responsible for phagocytosing foreign material^20^. Previous work within our group has shown microglia have the capacity to internalize and degrade nanosized materials^28^. Therefore, they are expected to be the principal cell type responsible for processing brain-penetrating AgNPs. Hence, understanding how microglia take up AgNPs and the mechanisms employed to process them will allow for the bioreactivity and biopersistence of AgNPs to be better predicted.

AgNP toxicity mainly arises from released Ag^+^ ions interacting with and damaging cell membranes, thiol protein groups and DNA^29^,^30^,^31^,^32^. Previous studies indicate silver nanowire toxicity may be limited by sequestration of released Ag^+^ ions through sulphiding reactions^33^. However, it is unknown whether Ag^+^ ion sulphidation is elicited by silver nanoparticles in microglial cells, and, if so, which mechanism is employed. With these questions in mind, this study employs the murine microglial N9 cell line and thoroughly characterized citrate-capped AgNPs to test the hypothesis that Ag^+^ ions released from AgNPs following endocytosis by microglial cells induce expression of H_2_S-synthesizing enzymes, leading to reprecipitation of silver ions as insoluble Ag_2_S, reducing the toxicity of the AgNPs. As H_2_S is a potent anti-inflammatory agent^34^, the effect of AgNPs on microglial inflammation is also examined by quantification of the pro-inflammatory factors ROS, NO and TNF-α. Furthermore, the dopaminergic neuronal cell line N27 is employed to examine whether modulation of microglial inflammation by AgNPs affects microglia-mediated neurotoxicity.

## Results

### Characterisation of AgNPs

**Figure 1:**
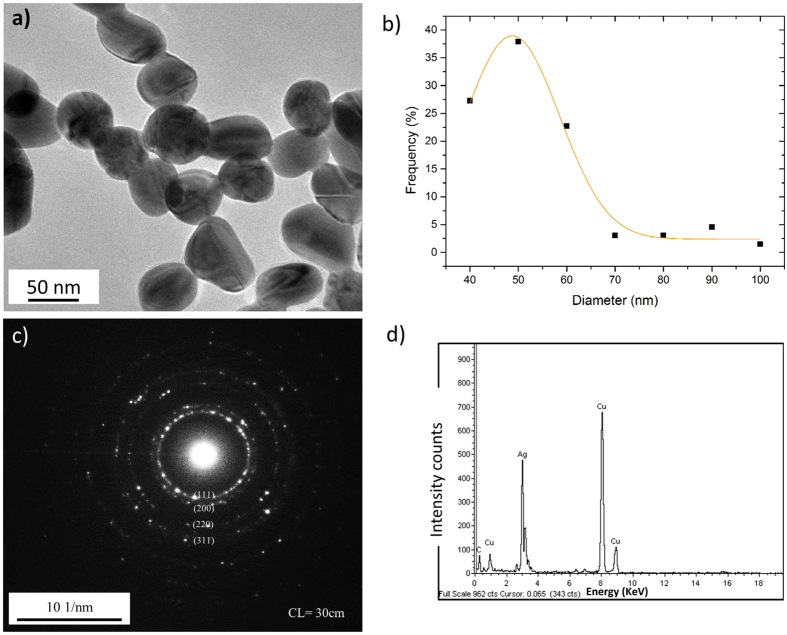
Characterization of the synthesised AgNPs. (**a**) TEM image of synthesised AgNPs and (**b**) their size distribution measured through TEM. (**c**) Indexed selected area electron diffraction (SAED) patterns of AgNPs incubated in DI water, revealing characteristic lattice spacings of 0.236, 0.204 and 0.145 nm, corresponding to the (111), (200) and (220) planes of metallic silver. The selective area aperture size used was 100 nm in diameter. (**d**) EDX spectra of AgNPs. Data shown are representative of analysis of 200 particles.

AgNPs were synthesised in-house and characterised using transmission electron microscopy (TEM), scanning transmission electron microscopy with energy-dispersive X ray spectroscopy (STEM-EDX), selected area electron diffraction (SAED), zeta (ζ)-potential and dynamic light scattering (DLS) analyses. Transmission electron microscopy showed AgNPs possessed uniform, spheroid morphologies, with a mean diameter of 49.7 ± 10.5 nm (Fig. 1a,b) (Table 1). SAED patterns (Fig. 1c) showed characteristic lattice spacings of 0.236 nm, 0.204 nm, and 0.145 nm, corresponding to the (111), (200) and (220) planes of metallic silver (see Table S2). EDX spectra acquired from the AgNPs (Fig. 1d) only identified silver from the sample, confirming both the successful removal of impurities following washing with DI water and that no adventitious sulphide was formed prior to cell exposure^35^. DLS measurements showed AgNPs aggregated into larger-sized particles after 1 hr incubation in RPMI cell culture medium (Table 2). The DLS measurement was consistent with ζ-potential measurements indicating AgNPs became less negative in RPMI at pH 7, compared to DI water (Table 2). The change in ζ-potential probably arises due to the screening effect of salts in cell culture medium on the surface charge of colloidal particles, which acts to decrease the electrostatic repulsive forces between particle surfaces, leading to aggregation of AgNPs^36^.

### Cellular uptake of AgNPs

AgNP internalization by N9 microglia was quantified through ICP-AES. Following a 1hr pulse- 24 hr chase AgNP (50 μg/mL) treatment, microglia internalized 29.96 ± 0.77% of the initial dose, with each individual cell internalizing an average of 30.52 ± 0.79 ng of silver (Table 3).

**Figure 2:**
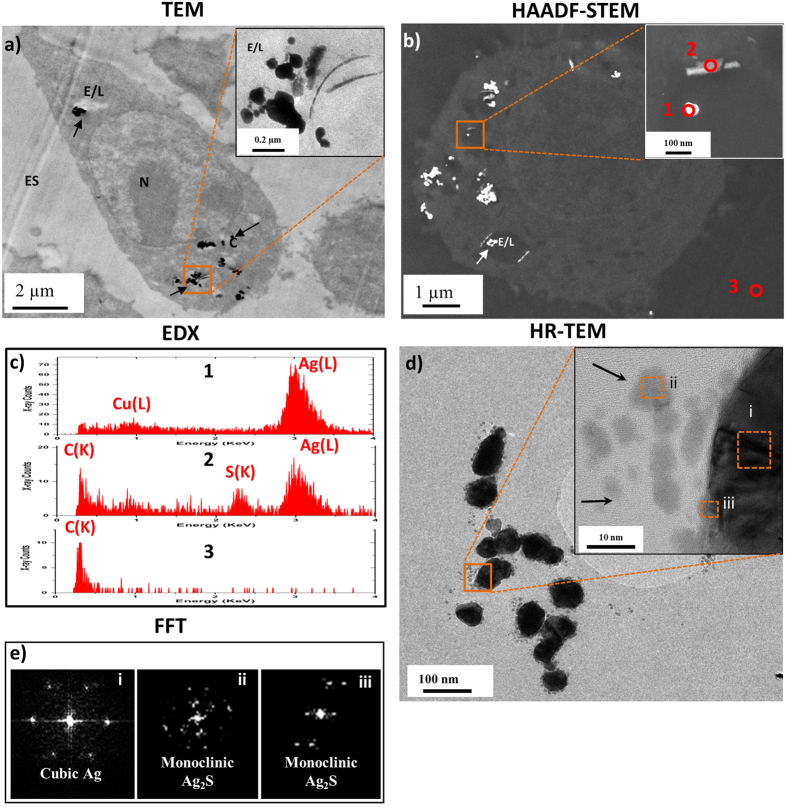
AgNP uptake and transformation by N9 microglia following a 1 hr pulse treatment (50 μg/mL) with 24 hrs chase period. (**a**) TEM images showing the cellular uptake of AgNPs by microglia cells (N: nucleus; C: cytoplasm; E/L: endosome/lysosome; ES: extracellular space). (**b**) HAADF-STEM images showing the intracellular distribution of AgNPs and a corresponding higher magnification image of the boxed area. (**c**) STEM-EDX spectra acquired from corresponding areas 1–3 marked in (**b**). (**d**) TEM imaging of intracellular AgNPs with secondary particles in close poximity to, and partially coating the surface of AgNPs. (**e**) FFT patterns taken from corresponding areas (i–iii) marked in (**d**) reveal secondary particles are composed of Ag_2_S. Images are representative of >100 cells surveyed per experiment, from three independent experiments.

Bright field (BF) TEM images confirmed microglial uptake of AgNPs after a 1 hr pulse treatment followed by a 24 hr chase period (Fig. 2a,b). AgNPs were observed within endosome/lysosome-like vesicles as well as within the cytoplasm (Fig. 2a,b, arrows). Some AgNPs agglomerated inside vesicles (Fig. 2a, insert), possibly due to the lower pH of the intravesicular environment and thermodynamic driving force to minimize total AgNP surface energy^37^.

The chemical and morphological changes of intracellular AgNPs were analyzed using high angle annular dark field (HAADF)-STEM and STEM–EDX (Fig. 2b,c). STEM-EDX spectra (Fig. 2c) taken from AgNPs and precipitates surrounding the intracellular AgNPs in Fig. 2b show Ag(L) peaks and S(K) peaks, indicating association of sulphur on or around the AgNPs. Phase contrast high resolution (HR) TEM images were acquired to examine whether the surface of the AgNPs had been fully or partially sulphided. To ensure sample preparation did not alter the physicochemistry of AgNPs inside the cells, the AgNP-exposed cells were prepared by a combination of high pressure freezing (HPF) and freeze substitution (FS). These methods induce the formation of amorphous ice that is subsequently replaced by organic solvents at low temperatures^38^, ensuring close-to-native preservation of cellular structures, AgNP composition and morphology, and ion distribution^39^. Formation of sub-particles on the surfaces of AgNPs was observed 24 hrs post-exposure, as indicated by their rough surfaces in HR-TEM analysis (Fig. 2d). Furthermore, fast fourier transform (FFT) patterns (Fig. 2e) from selected areas^40^ (inset Fig. 2d) revealed the sub-particles were composed of silver sulphide (Ag_2_S), probably due to Ag^+^ ions binding to organic ligands on the surface and vicinity of AgNPs, supporting the STEM-EDX observations. In contrast, the bulk of the AgNPs remained as pure silver. The interplanar spacing of the material coating the AgNP surface was measured from phase contrast HR-TEM images taken at the surface and the core of AgNPs. The spacing between the spots in the FFT pattern and their lattice plane identifications are shown in SI (Table S1).

TEM/STEM-EDX analysis of AgNPs incubated in RPMI (24 hrs) in the absence of cells revealed no structural or chemical modifications (Fig. S2), indicating no significant dissolution of AgNP and reprecipitation of Ag_2_S prior to cell internalization. Importantly, incubation of AgNP in perchlorate buffer (pH 5, corresponding to the average pH of endosomes) led to an increase in free Ag^+^ ions in solution at both 4 hrs and 24 hrs (Fig. S1), suggesting that the AgNP dissolution and subsequent Ag^+^ ion sulphidation requires cellular internalization.

### Effect of AgNPs internalization on H_2_S-synthesizing enzyme expression

Given the fact that H_2_S is a major sulphiding species for nanosilver^41^, and is an important regulator of cellular function, the effect of AgNPs on H_2_S synthesis was examined in order to elucidate the mechanism of AgNP sulphidation evidenced following internalization by microglia. H_2_S synthesis was indirectly measured by quantifying the expression of H_2_S-synthesizing enzymes.

#### Confocal Analysis

**Figure 3:**
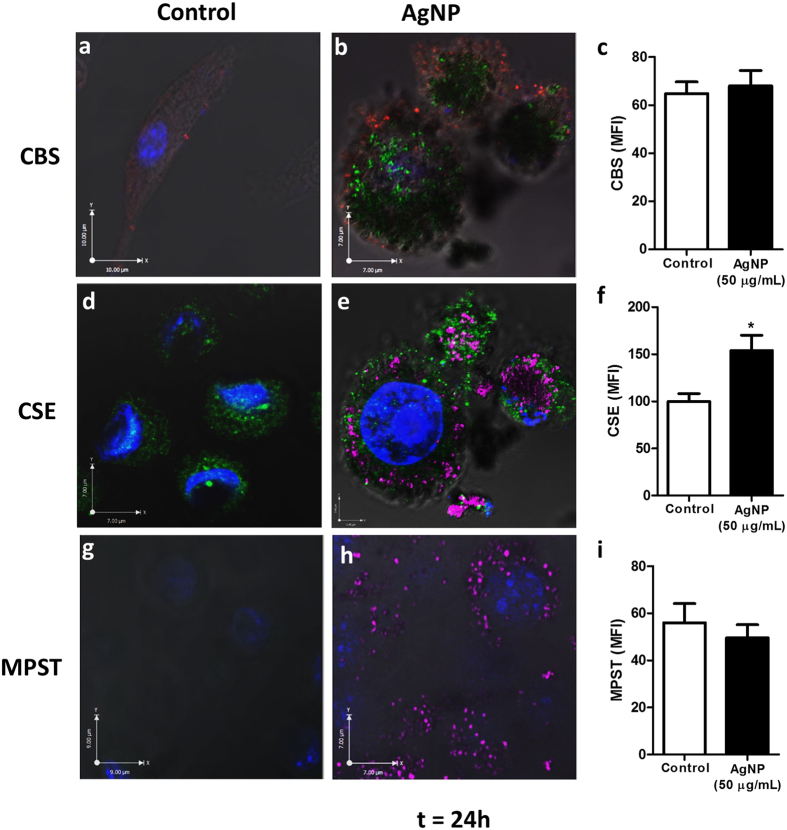
Cellular uptake and distribution of AgNPs combined with CBS, CSE and MPST enzymes expression in microglia. N9 microglia were treated with AgNP (50 μg/mL) for 1 hr (pulse) followed by a 24 hrs chase period. Cells were immunostained and enzyme expression analysed through confocal microscopy. Confocal images of CBS (**a**,**b**), CSE (**d**,**e**) and MPST (**g**,**h**) enzymes in control (**a**,**d**,**g**) and AgNP-treated (**b**,**e**,**h**) microglial cells. For images (**a**,**b**), CBS = red; AgNP = green; DAPI = blue. For images (**d**,**e**,**g**,**h**) CSE/MPST = green, AgNP = magenta, DAPI = blue. White = colocalization of enzymes and AgNP. Images are representative of three independent experiments. The mean fluorescent intensity (MFI) of CBS (**c**), CSE (**f**) and MPST (**i**) immunoreactivity was measured from the confocal microscopy images using Image J analysis software. Results are displayed as mean ± SD of three independent experiments, quantifying 40 individual cells per experiment. *Denotes *p* < 0.05 *vs*. control, as assessed by a student’s t-test.

The expression of the H_2_S-synthesizing enzymes cystathionine-γ-lyase (CSE), cystathionine β-synthase (CBS) and mercaptopyruvate sulfurtransferase (MPST) was examined by confocal microscopy after a 1 hr pulse AgNP treatment followed by a 24 hrs chase period. Confocal microscopy images indicated baseline enzyme expression in control cells was stronger for CSE compared to CBS and MPST (Fig. 3a,d,g), suggesting CSE is a major H_2_S producer in microglial cells. A similar expression pattern was seen at an earlier time-point (4 hrs chase period) (Fig. S3).

Treatment of microglia with AgNPs led to an increase in CSE signal intensity in the vicinity of AgNPs (Fig. 3d,e), without affecting CBS signal intensity (Fig. 3a,b). MPST was weakly detected in both control and AgNP-treated cells (Fig. 3g,h). Quantitative analysis of the confocal images revealed that the CSE enzyme level was significantly increased following exposure to AgNPs, with a 50% mean fluorescence intensity increase observed compared to control cells (Fig. 3f). However, neither CBS (Fig. 3c) nor MPST (Fig. 3i) enzyme levels were significantly affected by AgNP internalization.

#### Western blot immunodetection

**Figure 4:**
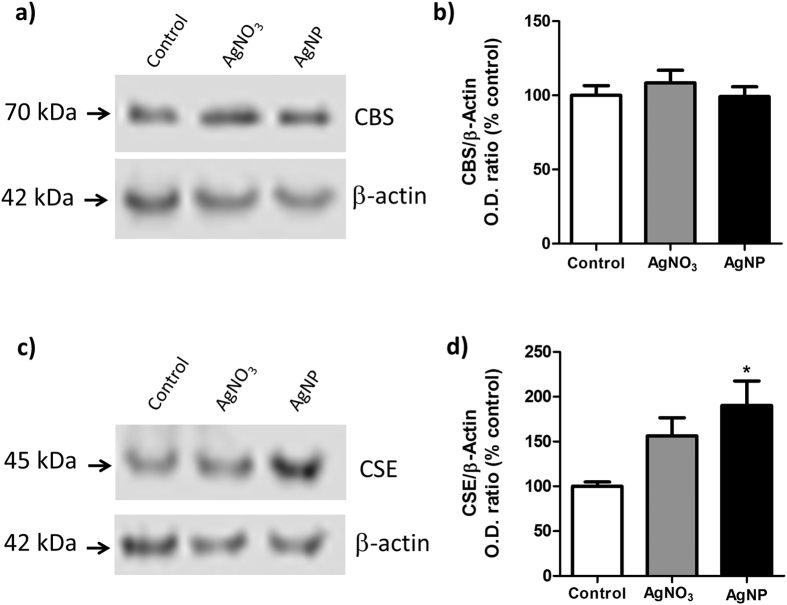
Modulation of microglial CBS and CSE expression by AgNP or AgNO_3_ treatment. N9 microglia were treated with AgNP (50 μg/mL) or AgNO_3_ (3.5 μM) for 1 hr (pulse) followed by a 24 hrs chase period. CBS/CSE protein expression was then quantified by immunostaining Western blots from whole cell lysates. Immunostaining for β-actin was used as loading control. Image in (**a**,**c**) is a representative Western blot from three independent experiments, from which enzyme expression was quantified by normalizing enzyme optical density against β-actin optical density (**b**,**d**). *Denotes *p* < 0.05 *vs*. control, as assessed by a one-way ANOVA. Results are displayed as mean ± SEM of three independent experiments.

To confirm the enzyme expression results obtained through confocal microscopy analysis, CBS and CSE enzyme expression levels were quantified through Western blotting. MPST was omitted from the WB analysis due to its negligible detection through confocal microscopy for all conditions and time-points. As shown in Fig. 4, Western blot data indicated CBS expression remained unaltered following AgNP treatment (1 hr pulse exposure with 24 hrs chase) (Fig. 4a,b), while expression of CSE was significantly increased (Fig. 4c,d), consistent with the confocal analysis images. In order to verify the modulation of enzyme expression was due to Ag^+^ ions released from AgNPs, microglia were subjected to a similar pulse-chase treatment with silver nitrate (AgNO_3_) as a positive control for the presence of silver ions. Consistent with the effects of AgNP treatment, CBS expression was not affected by AgNO_3_ treatment (Fig. 4a,b). While expression of CSE appears upregulated by AgNO_3_ treatment, the change marginally failed to reach statistical significance (Fig. 4c,d).

### Effect of AgNP treatment on microglial inflammation and cell viability

**Figure 5:**
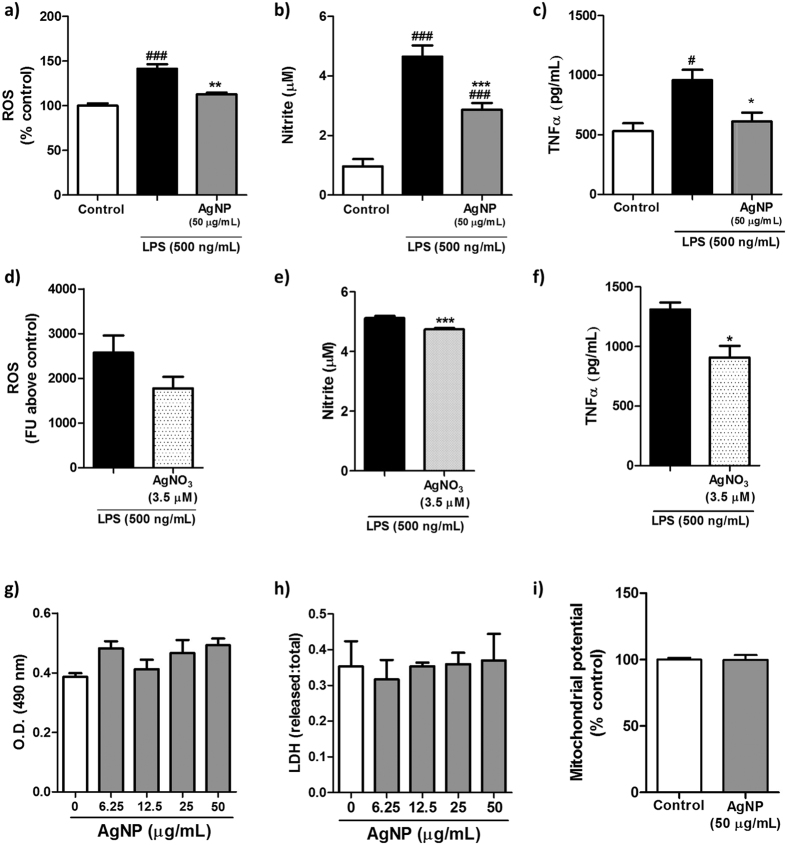
Modulation of microglial reactivity and cell viability by AgNP/AgNO_3_ treatment. N9 microglial cells were treated with LPS (500 ng/mL) with or without AgNPs/AgNO_3_ for a 1 hr pulse period followed by a 24 hrs chase period. Microglial inflammation was assessed through quantification of (**a**,**d**) ROS production, (**b**,**e**) nitrite production, and (**c**,**f**) TNFα release. Cell viability was assessed through (**g**) an MTS assay, (**h**) LDH release assay, and (**i**) mitochondrial potential quantification. Data is presented as mean ± SEM of three independent experiments; #, ### indicate *p* < 0.05, 0.005, respectively, *vs*. control; *, ** and *** indicate *p* < 0.05, 0.01, 0.005, respectively, *vs*. LPS treatment as assessed by a one-way ANOVA with Tukey’s *post-hoc* test (**a**,**b**,**c**,**g**,**h**) or a student’s t-test (**d**,**e**,**f**,**i**).

Given H_2_S is a known important modulator of inflammation, especially in microglia and macrophages^42^,^43^, the effect of AgNP treatment on microglial inflammation was examined by quantifying its effects on LPS-induced ROS production, nitrite synthesis and TNFα release. A simultaneous 1 hr treatment of microglia with LPS (500 ng/mL) and AgNPs followed by a 24 hrs chase period led to an overall reduction in pro-inflammatory microglial markers, as evidenced by a reduction in ROS production (Fig. 5a), nitrite synthesis (Fig. 5b) and TNFα release (Fig. 5c) compared to LPS treatment alone. Consistent with the CSE enzyme expression data, the anti-inflammatory effects of AgNPs were mirrored by a similar treatment with AgNO_3_ (Fig. 5d–f), however the effects were less pronounced, and in the case of ROS reduction did not reach statistical significance (Fig. 5d).

Importantly, the effects of AgNPs/AgNO_3_ on ROS production, TNFα release and nitrite synthesis were due to a *bona fide* anti-inflammatory effect and not a decrease in cell viability, as evidenced by a lack of a cytotoxic effect by either AgNPs (Fig. 5g–i) or AgNO_3_ (Fig. S4). Interestingly, a similar AgNP treatment of N27 dopaminergic neurons induced significant neurotoxicity (Fig. S5), supporting silver ion sulphidation as a detoxifying mechanism inside microglial cells.

### Effect of AgNP treatment on microglia-mediated neurotoxicity

**Figure 6:**
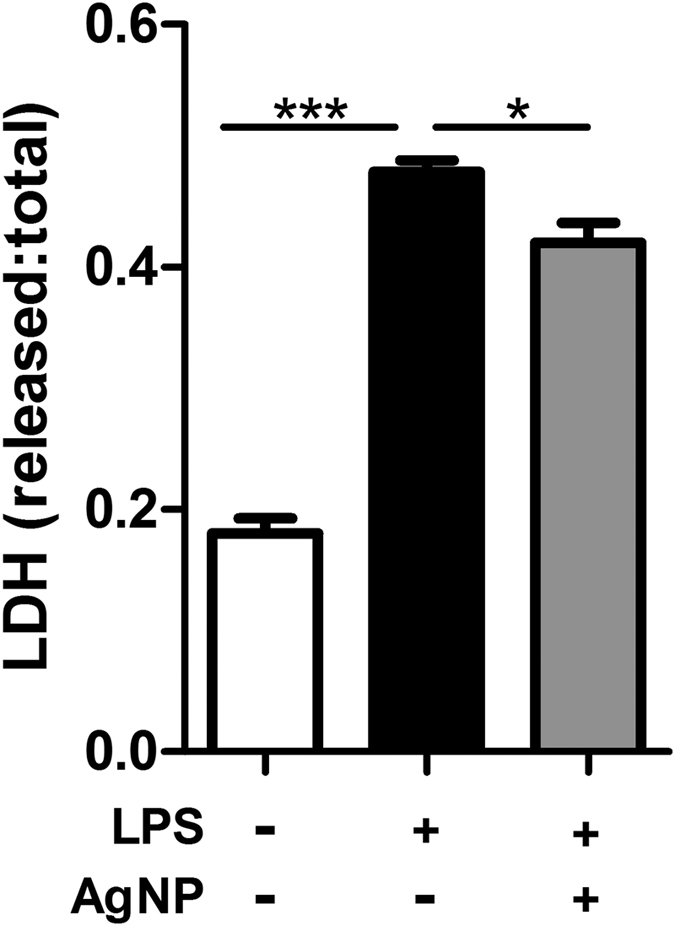
Modulation of microglial neurotoxicity by AgNP treatment. N9 microglia were treated for 1 hr (pulse) with AgNP (50 μg/mL) and/or LPS (500 ng/mL), followed by a 24 hrs chase period with only LPS present. The medium was then transferred to N27 neurons and incubated for 48 hrs, after which time-point cell viability was assessed through an LDH release assay. Data is presented as mean ± SEM of three independent experiments; * and *** indicate *p* < 0.05, 0.005, respectively, *vs*. corresponding groups, as assessed by a one-way ANOVA with Tukey’s *post-hoc* test.

In order to examine whether the reduction of microglial inflammation by AgNP treatment translated into modulation of neurotoxicity, dopaminergic N27 neurons were incubated (48 hr) with microglial medium from control, LPS or LPS/AgNP-treated microglia. LPS treatment induced a significant neurotoxic microglial phenotype, as evidenced by a robust increase in LDH release from neurons incubated with medium derived from LPS-treated microglia, compared to neurons incubated in medium derived from control microglia (Fig. 6). Consistent with the reduction in microglial inflammation by AgNPs, co-treatment of N9 microglia with AgNP reduced the LPS-induced neurotoxicity, as evidenced by decreased LDH release from neurons incubated in medium derived from LPS/AgNP-treated microglia compared to neurons incubated in medium derived from microglia treated with LPS alone (Fig. 6). In order to corroborate the reduction of microglia-mediated neurotoxicity by AgNP treatment, neuronal viability was further assessed by quantifying metabolic activity through an MTS assay following microglia treatment with commercially acquired AgNP (see Supplementary Materials and methods). In agreement with the LDH release results, AgNP treatment of microglia abolished the LPS-induced microglia neurotoxicity, as evidenced by statistically equal levels of metabolic activity of neurons incubated with medium derived from control microglia *vs*. LPS/AgNP-treated microglia (Fig. S6a). Importantly, treatment of microglia with commercially acquired AgNP reduced LPS-induced microglial inflammation (Fig. S6b) similarly to the in-house synthezised AgNP.

## Discussion

In the present study, we directly assessed the internalization of AgNPs into a mouse microglial cell line, examining the intracellular chemical and morphological transformation of the nanoparticles, as well as the effect of AgNP-treatment on microglial viability and LPS-induced microglial inflammation and related neurotoxicity.

ICP-AES and TEM imaging clearly showed AgNPs internalization by microglial cells following a 1 hr exposure. Furthermore, STEM-EDX spectra and FFT patterns indicated Ag_2_S complexes formed intracellularly in the vicinity of AgNPs, as well as partially coating the nanoparticles. As internalized AgNPs are not thermodynamically stable in the acidic and oxidising intracellular environment of endosomes, Ag^+^ ions are released from the AgNPs due to nanoparticle dissolution. The Ag^+^ ions react with sulphide species to form the evidenced Ag_2_S complexes. Since the solubility of Ag_2_S is extremely low (K_sp_ = 5.92 × 10^−51^), AgNP-surface coating by Ag_2_S complexes would modify the surface properties of AgNPs and inhibit further oxidative dissolution of AgNP^33^. The formation of Ag_2_S as a Ag^+^ sequestering mechanism is expected to occur in preference over formation of silver chloride and silver oxide, as their solubility is significantly higher than that of Ag_2_S (K_sp_ = 1.77 × 10^−10^ and 4 × 10^−11^, respectively)^33^, and therefore they will not be thermodynamically favoured.

AgNP cytotoxicity is mainly due to the release of Ag^+^ ions, which interact via several damaging mechanisms: Ag^+^ ions interact with cell membranes, leading to lower membrane integrity and increased permeability; they are able to bind thiol groups in proteins, causing improper protein function; and they bind to and damage DNA, resulting in cell apoptosis^29^,^30^,^31^,^32^. Recent studies indicate that the transformation of Ag^+^ ions to insoluble Ag_2_S reduces the potential toxicity of silver nanomaterials in the lung^33^. Therefore, the formation of Ag_2_S complexes around AgNPs may represent a Ag^+^-sequestering and detoxifying mechanism similarly present in microglial cells. Such a mechanism would explain the lack of AgNP-induced cell death evidenced in the current study, as a similar concentration of AgNPs induced significant toxicity to dopaminergic neurons in the present study, and has been shown to be toxic to other cell types^14^,^44^.

Cell type differences in H_2_S levels and/or in the ability to up-regulate H_2_S-synthesizing enzyme expression would affect the cell’s capacity to sulphide and sequester released Ag^+^ ions, hence may underlie the differing susceptibility of cells to AgNP-induced toxicity. H_2_S is produced mainly by the pyridoxal-5′-phosphate (PLP)-dependent enzymes cystathionine β-synthase (CBS) and cystathionine γ-lyase (CSE) *via* the amino acids homocysteine, cysteine and cystathionine^45^,^46^, as well as by 3-mercaptopyruvate sulfurtransferase (MPST) *via* the cysteine catabolic pathway^47^. We have demonstrated the H_2_S-synthesizing enzyme CSE becomes up-regulated in response to the sulphur-sequestering capacity of Ag^+^, while the alternative H_2_S-synthesizing enzymes CBS and MPST remained unchanged. Indeed, the levels of H_2_S have been shown to be able to regulate both the expression and activity rate of CSE in mammalian cells and tissue^48^,^49^. Hence, a decrease in available H_2_S following Ag^+^ sequestration would prompt the up-regulation of CSE to maintain H_2_S homeostasis. This process might not be present in neurons, resulting in insufficient H_2_S levels necessary for efficient AgNP detoxification. Indeed, expression of hydrogen sulphide-producing enzymes has been shown to be higher in glial cells compared to neuronal cells^50^,^51^. Moreover, certain neuronal populations have been shown to lack CSE as a primary H_2_S synthesising enzyme^52^. Therefore, lack of CSE may restrict the ability of certain neuronal populations to rapidly counteract the decreasing H_2_S levels, preventing detoxification of Ag^+^. In addition, given that microglia are the sentinels of the CNS, they are programmed to actively respond to infiltrating material within their extracellular environment. Hence, the presence of intracellular AgNPs may elicit an active response involving localisation of CSE and H_2_S synthesis in the vicinity of the nanoparticles, as opposed to passive sulphiding of Ag^+^ ions by available H_2_S. Thus, Ag^+^ ions released within microglia will be sulphided and sequestered more efficiently in comparison to neurons, limiting their toxicity towards microglial cells. Speculatively, given CSE is expressed by a number of different tissues both centrally and peripherally^53^,^54^, its up-regulation by AgNPs might be a general mechanism of silver toxicity limitation in a wide range of tissues.

The sulphiding of AgNPs is a complex process which may occur *via* two chemical pathways, depending on the sulphide concentration in the cellular environment. According to Liu *et al*., Ag sulphiding is dominated by an indirect reaction if the sulphide concentration is <0.025 μg/mL^41^. At higher sulphide concentrations, the mechanism switches to a direct reaction where the reaction rate increases with increasing sulphide concentration. The reactions below show the two competing chemical and transport pathways of AgNP sulphiding before transformation into the thermodynamically stable sulphide phases:

1. Direct route (particle-fluid heterogeneous reaction) - AgNPs direct oxysulphidation reaction rate increases with increasing sulphide concentration.
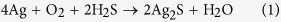

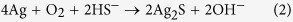

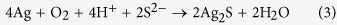
2. Indirect route (oxidative dissolution (Equation 4) and precipitation (Equations 5,6,7)) - dissolution of AgNPs is initiated by oxygen chemisorption accompanied by electron transfer^37^. The cooperative effect of both dissolved oxygen and protons by thermodynamic analysis can be represented in the following heterogeneous oxidation reaction stoichiometry:

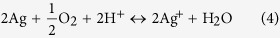


While Ag^+^ ions are released from AgNPs, they rapidly diffuse into the cellular environment and form sulphide by subsequent precipitation:


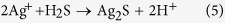



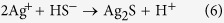



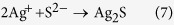


Initially, as intracellular sulphide concentration is maximum, sulphide species (*e*.*g*. H_2_S, HS^−^ and S_2_^−^) may attack the outer layer of AgNPs surface *via* a direct particle-fluid reaction, leading to the formation of small Ag_2_S particles in the vicinity of the NPs surface, as observed through HR-TEM. Over time, released Ag^+^ ions will either deposit on the existing Ag_2_S nuclei to form larger-sized Ag_2_S particles or precipitate as new Ag_2_S particles. It will be interesting to examine how microglia process the accumulating Ag_2_S at longer time-points.

Nanomaterials have been shown to be able to induce cell death through a recently characterized non-apoptotic mechanism involving iron and lipid peroxidation termed ferroptosis^55^,^56^. While microglia in the current study are resistant to AgNP-induced ferroptosis as assessed by lack of membrane integrity and metabolic activity decrease, as well as the lack of ROS production increase (essential for the lipid-peroxidation mediated cell death), it will be of interest to examine whether AgNP are able to induce ferroptosis in neuronal cells and whether it is the main mechanism responsible for the differing susceptibilities of different cell types to AgNP-induced toxicity.

In the present study, increased expression of the H_2_S-synthesising enzyme CSE following AgNP treatment, in addition to correlating with Ag^+^ sulphide deposition and reduced AgNP toxicity, correlated with decreased microglial inflammation. Indeed, there is a growing body of evidence indicating H_2_S regulates inflammation^42^,^57^, and our results echo previous studies examining the role of H_2_S-synthesizing enzymes in modulation of microglial reactivity. For instance, treatment of microglia with the neurotoxic compound rotenone leads to an increase in microglial inflammation with a concomitant reduction in the levels of the H_2_S-synthesizing enzyme CBS^58^. Importantly, if CBS expression or H_2_S levels were enhanced, rotenone-induced microglial reactivity was decreased. Similarly, treatment of peripheral macrophages with oxidized low density lipoprotein (oxLDL) leads to a reduction in the expression levels of CSE with a concomitant increase in TNFα production which can be prevented by overexpression of CSE or exogenous H_2_S^59^. Importantly, CSE expression has also been detected in primary microglia^60^. Therefore, the present results demonstrate the increase in CSE expression to counterbalance the decreasing H_2_S levels due to Ag^+^ sequestration confers AgNPs with an anti-inflammatory effect. Furthermore, this anti-inflammatory effect translated into reduced microglia-mediated neurotoxicity. This finding has important consequences for AgNP-induced neurotoxicity, as microglial inflammation is known to contribute to neuronal toxicity, especially in cases of chronic neurodegeneration such as during PD^61^. Hence, from the results presented in this paper, it may be surmised that *in vivo* AgNP neurotoxicity is due to direct neuronal effects of AgNPs and not due to exacerbation of microglia-derived neurotoxic factors. Speculatively, highly controlled targeting of AgNP into microglia could decrease brain inflammation locally by inhibiting microglia reactivity through promotion of H_2_S synthesis, thereby reducing inflammatory injury to neighbouring neuronal cells. Additionally, since H_2_S has been shown to be a potent neuroprotective agent in models of neurodegenerative diseases^62^, induction of H_2_S synthesis by microglial targeted AgNPs could actually aid in decreasing chronic neurodegeneration. Interestingly, recent experiments have demonstrated a deficiency in CSE activity in human brain tissue derived from Huntington’s disease patients, which may underlie the progressive neurodegeneration seen in that disease^63^.

In summary, the present study proposes a AgNP-detoxifying mechanism in microglia involving the sulphiding of Ag^+^ ions and up-regulation of H_2_S-synthesizing enzymes. Importantly, this study demonstrates for the first time a correlation between AgNP-detoxifying mechanisms and a reduction in microglial inflammation which serves a neuroprotective role. In addition, it highlights the need for further characterisation of AgNPs at the cellular level inside brain tissue and other target organs to assess how AgNP transformation varies between relevant cell types and AgNP formats with varied physicochemistry, and thus how it affects AgNP bioreactivity and biopersistence. Future studies in experimental animal models may likewise benefit from the application of these methods to determine whether AgNP transformation processes occur *in vivo*. Characterisation of cellular uptake and transformation of AgNP may have important implications for understanding and ultimately providing guidelines to control the biopersistence and bioreactivity of AgNP which can be used to make informed decisions about how to design future classes of “safe” AgNPs.

## Materials and Methods

### Nanoparticle synthesis, characterisation and stability

#### Silver nanoparticle synthesis

Silver nanoparticles were synthesised *via* the chemical bath reduction method in which trisodium citrate (Na_3_C_6_H_5_O_7_) (Fisher Scientific, UK) serves a dual role as both reductant and stabilizer. Briefly, silver nitrite (AgNO_3_) (1.0 × 10^−3^ M) (Sigma-Aldrich, UK) solution was added to preheated de-ionized (DI) water (180 mL). Then, Na_3_C_6_H_5_O_7_ (1.0 × 10^−3^ M) solution was added dropwise to the AgNO_3_ solution as soon as boiling commenced. The color of the solution slowly turned grayish yellow, indicating the reduction of Ag^+^ ions. The solution was heated continuously for an additional 20 min, and then cooled to room temperature. In order to remove impurities and excess citrate, the AgNPs suspension was washed with DI water and centrifuged at a relative centrifugal force maximum value (RCF_max_) of 10,000 g. This washing process was repeated three times to ensure no citrate remained in the solutions. Finally, citrate-coated AgNPs were suspended in DI water at the desired concentration, sealed and stored in the dark at 4 °C. The purity of synthesized AgNPs was confirmed using an energy dispersive X-ray (EDX) spectrometer mounted on a LEO Gemini 1525 field emission gun scanning electron microscope (FEGSEM), to ensure that AgNPs were not sulphided by adventitious contaminants in the atmosphere^64^ and that impurities, such as Na^+^ and Cl^−^ ions had been removed by the washing process.

The size and surface charge of AgNPs in water, RPMI (1 hr) and DMEM (24 hrs) were measured using dynamic light scattering (DLS) and zeta potential analyses (Malvern Zetasizer Nano series), respectively.

#### Stability of AgNPs: Dissolution Kinetics

The amount of Ag dissolved from AgNPs in pH 5 perchlorate buffer solutions, corresponding to the pH found in intracellular endosomes, was analysed using inductively coupled plasma-atomic emission spectroscopy (ICP-AES). Non-interacting buffer solutions were used to minimise the effect of anions on the stability of Ag^+^ ions. The AgNP suspensions were incubated in perchlorate buffer solutions at 37 °C for 4 or 24 hrs followed by centrifugation with 2 kDa (<4 nm) filter tubes (Sartorius Stedim VIVACON 500) at 13,000 rpm to separate particles from the solutions. Chen *et al*.^35^ showed that dissolved Ag^+^ ions will reprecipitate as insoluble compounds in cell culture media (RPMI and DMEM), therefore ICP-AES cannot be employed to quantify the release of Ag^+^ ions in the medium.

### Cell culture and treatments

#### N9 microglial cells

Microglia experiments were carried out on the immortalized embryonic mouse microglia N9 cell line, first developed by Dr. Ricciardi-Castagnoli *et al*.^65^ and given as a kind gift by Dr. Deanna Taylor, Imperial College London. N9 microglia reliably replicate cultured primary microglia with respect to NO production, cytokine synthesis and expression of cell surface markers^66^,^67^,^68^. N9 microglia were cultured in Dulbecco’s Modified Eagle’s Medium (DMEM, Sigma-Aldrich, UK) with 5% Fetal Calf Serum (FCS, Sigma-Aldrich, UK), 8 mM L-glutamine (Sigma-Aldrich, UK) and 50 U/mL Penicillin and 50 μg/mL streptomycin (Sigma-Aldrich, UK) (termed full DMEM) at 37 °C in a humidified atmosphere with 5% CO_2_.

Microglial cells were plated either in 96 well plates, onto glass coverslips in 24-well plates, or 6 well plates (at 10,000, 50,000 or 500,000 cells per well, respectively) and incubated for 24 hrs before experimentation began to allow them to readopt a resting phenotype. Microglia were then washed with serum-free (SF) RPMI medium (Sigma-Aldrich, UK) (with 8 mM L-glutamine and 50 U/mL Penicillin and 50 μg/mL streptomycin; termed SF-RPMI) and incubated with AgNP alone, lipopolysaccharide (LPS, 500 ng/mL^28^) (Enzo Life Sciences, UK) alone or a combination of both in SF-RPMI for 1 hr (pulse) at 37 °C. The cells were then washed with SF-RPMI and incubated in SF-RPMI for a further hour. Finally, cells were incubated in full DMEM for 4 or 24 hrs (chase) before analysis (*i*.*e*. experimental end-point). SF-RPMI medium was used during AgNP exposure as AgNP do not dissolve or transform to Ag_2_S in this cell culture medium^33^. Cells were then incubated in full DMEM to ensure appropriate metabolic support.

#### N27 neuronal cells

Neuronal experiments were carried out on the immortalized foetal rat ventral mesencephalon dopaminergic N27 cell line, used extensively as an *in vitro* PD model^69^,^70^,^71^. N27 cells were a kind gift from Dr. Nabil Hajji, Imperial College London. Neuronal cells were cultured in full DMEM at 37 °C in a humidified atmosphere with 5% CO_2_. For experimentation, cells were removed from culture flask through trypsinization and plated in 12 well plates in full DMEM 24 hrs prior to experimentation to ensure cell attachment.

For microglia-mediated neurotoxicity studies, N9 microglia plated in 6 well plates were treated with LPS with or without AgNP as above (see ‘cell culture and treatment’ section). However, in order to ensure sufficient microglial activation to induce neuronal cell death, LPS was present in the cell culture medium throughout the 24 hrs chase period. At the experimental end-point, the medium was collected and centrifuged (5 min at × 1200 g) to clear floating cells. The supernatant was then transferred to the cultured N27 neurons and incubated for 48 hrs prior to cell viability quantification.

### Cell viability assays

#### MTS assay

MTS is a chromogenic assay which involves the bioreduction of the tetrazolium compound 3-(4,5-dimethylthiazol-2-yl)-5-(3-carboxymethoxyphenyl)-2-(4-sulfophenyl)-2H-tetrazolium by NADH in viable cells into a coloured formazan product. Therefore, the cellular metabolic activity can be quantified spectrophotometrically by measuring the absorbance of the formazan product.

At the experimental end-point, cells plated in 96 well plates were washed and incubated at 37 °C with fresh full DMEM (100 μL) containing MTS reagent (20 μL) (Promega, UK) for 2–3 hr before the optical density (at 490 nm) was quantified with a plate reader. Cell viability was determined by comparing the optical density of treated cells *vs*. control cells.

#### LDH assay

Lactate dehydrogenase (LDH) is a soluble cytosolic enzyme which catalyses the oxidation of lactate to pyruvate. Once cells lose their membrane integrity due to stress or injury, LDH is rapidly released into the extracellular medium. Thus, membrane damage and cytotoxicity can be assessed by the quantification of released LDH from cells.

At the experimental end-point, extracellular medium (50 μL) from cells plated in 24 well plates was incubated with LDH detection solution (50 μL) (Promega, UK). Following 10–20 min incubation (at 37 °C), the optical density (at 490 nm) was quantified with a plate reader to determine LDH concentration in the extracellular medium. To control for lower LDH in the extracellular medium due to a sparser cell population following cytotoxicity, total LDH (*i*.*e*. intracellular plus released LDH) was also quantified by lysing cells in the original culture medium (with 10 μL lysing buffer, Promega, UK) and the cell medium incubated with LDH reaction mixture as before. The amount of released LDH was then normalized against the total amount of LDH in the culture well.

### Cellular uptake of AgNPs

N9 cells were exposed to 50 μg/mL AgNPs as above. This high dose has been shown to be toxic to neurons *in vitro*^12^,^14^, but showed no toxicity to microglia in the present study, therefore was suitable to provide evidence that Ag_2_S formation acts as a detoxifying mechanism for silver ions released from intracellular AgNPs.

#### Transmission electron microscopy

N9 microglia were rinsed with HEPES buffer and fixed in freshly prepared 2.5% glutaraldehyde for 1 hr at 4 °C. Each sample was dehydrated in a graded ethanol series (50%, 70%, 90% and dry100% volume ratio of ethanol to DI water) for 5 min × 3 times each, followed by dry acetronitrile (Sigma-Aldrich, UK) for an additional 10 min each, all at room temperature. After dehydration, samples were progressively infiltrated with 50% and 75% Quetol-based araldite resin/ethanol solutions (Agar scientific, UK) overnight and finally 100% resin for 4 days with fresh resin replaced daily. The specimens were then cured at 60 °C for 24 hrs before sectioning using an ultramicrotome (Leica ultracut UCT, Wien, Austria). Ultrathin cell monolayers (approximately 50–100 nm) were cut with a 35° diamond knife and each section was examined in a JEOL 2000 transmission electron microscope at an accelerating voltage of 80 kV. The use of heavy metal stains to enhance cell ultrastructure in the TEM can alter the morphology and accelerate the oxidation of AgNPs^72^, and therefore was avoided. Our previous published work has shown that the sample preparation procedure applied in this study does not alter the physicochemistry of the AgNPs in the cellular environment^72^.

Multiple cells (>100 per sample) from three different cell exposures were surveyed using an FEI Titan 80–300 scanning/transmission electron microscope operated at 80 kV, fitted with a Cs (image) corrector. HAADF-STEM was performed with a convergence semi-angle of 14 mrad and inner and outer collection semi-angles of 49 and 239 mrad, respectively. The probe diameter was <0.5 nm. STEM-EDX spectroscopy was performed using a SiLi EDX spectrometer (EDAX, Leicester UK).

#### High-pressure freezing

High-pressure freezing (HPS)/freeze substitution (FS) was employed to ensure preservation of cellular structures, AgNP composition and ion distribution. Samples were placed in specimen carriers with 200 μm deep cavities, mounted in a 20% (wt/vol) solution of bovine serum albumin (Sigma) in αMEM, and frozen using a Leica EM PACT 2 (Leica Microsystems) high-pressure freezer. FS was performed in a Leica electron microscope AFS2 freeze substitution device using an anhydrous solution of acetone containing a 3% (vol/vol) glutaldehyde, 1% (wt/vol) osmium tetraoxide, and 0.5% (wt/vol) uranyl acetate at −90 °C for 8 h. Samples were then gradually warmed to 0 °C at 5 °C/h, washed twice in acetone, brought to room temperature, and infiltrated with resin, as described in the TEM methodology section.

#### Inductively-coupled plasma atomic emission spectroscopy

The amount of silver internalized by N9 microglia following AgNP treatment (see above) was quantified through inductively-coupled plasma atomic emission spectroscopy (ICP-AES). At the experimental end-point, the cells were rinsed twice with 1 mL HBSS to remove all non-internalized silver. Following collection of the cells in 1 mL HBSS, cell number was quantified with the use of a haemocytometer. The cells were then pelleted by centrifugation (10,000 g × 10 mins) and resuspended in 200 μL HBSS. The cell suspension was digested with 800 μL aqua regia overnight and neutralized with 3 mL diH_2_O before ICP-AES silver quantification. AgNP internalization was calculated as a percentage of initial dose or total silver internalization per cell.

### Microglial reactivity quantification

#### TNFα release

At the experimental end-point, TNFα release was measured in the cell culture medium using commercially available ELISA kits (Peprotech, UK) according to the manufacturer’s instruction.

#### Intracellular reactive oxygen species (ROS) production

Intracellular ROS levels were detected using the 2′,7′-Dichlorofluorescin diacetate (DCFH-DA) assay (Sigma, UK). DCFH-DA is a lipophilic cell permeable compound that is deacetylated in the cytoplasm by cellular esterases to 2′, 7′-dichlorofluorescein (DCF), which is then oxidised by free radicals, including hydroxyl, alkoxyl, peroxyl, carbonate and nitrate radicals to produce a fluorescence signal.

For ROS quantification, N9 microglia were seeded in dark, flat transparent bottomed 96 well plates (10,000 cells per well) as above (see ‘cell culture and treatment’ section). At the experimental end-point, cell media was removed and replaced with 25 μM DCFDA diluted in full DMEM without phenol red for 45 min at 37 °C protected from light. Cells were then washed twice with PBS before measurement of fluorescence (at emission 485 nm and excitation 535 nm) in a microplate reader.

#### Nitric Oxide production

Due to its short half-life, production of nitric oxide (NO) was assessed through quantification of its stable metabolite nitrite (NO^−^_2_) using the Griess reaction^73^. At the experimental end-point, 75 μl of cell culture medium was transferred to 96 well plates and incubated with 75 μL of Griess reagent (sulfonilamide plus 1-(naphthyl)ethylenediamine) (Sigma-Aldrich, UK) at RT in the dark. Levels of nitrite were quantified by measuring absorbance at a wavelength of 540 nm after 5–10 min incubation. Optical density was converted to nitrite concentration using a sodium nitrite standard curve.

#### Mitochondria membrane potential

Mitochondrial membrane potential (MMP) was determined using the red-orange fluorescent dye tetramethylrhodamine ethyl ester perchlorate (TMRE) (Sigma, UK). This lipophilic, positively-charged dye enters cells and is sequestered by healthy mitochondria with a negative charge. Conversely, depolarized or inactive mitochondria lose their membrane potential, leading to a loss of their negative charge and ability to sequester TMRE. Therefore, the polarization state of mitochondria can be assessed by the amount of orange-red fluorescence inside cells.

At the experimental end-point, cells in 96 well plates were rinsed twice in PBS and incubated in 500 nM TMRE for 20 mins at 37 °C in the dark, followed by rinsing with 0.2% bovine serum albumin (BSA) in PBS prior to fluorescence measurement with excitation at 549 nm and emission at 575 nm using a microplate reader.

### Enzyme expression quantification

#### Western Blotting

Protein expression was quantified through immunostaining of Western blots (WB) from N9 lysates. Cells seeded in 6 well plates were lysed with RIPA buffer (Sigma, UK) with 10% protein inhibitor cocktail (Aprotinin, Bestatin, E-64, Leupeptin, Pepstatin, AEBSF) (Sigma, UK) at the experimental end-point. The lysates were cleared by centrifugation and 20 μg of protein resolved by SDS-PAGE (10% gel) and transferred to PVDF membranes. After blocking with 5% BSA in TBS-T (2 hr at room temperature), the WB were stained with anti-CBS (40 ng/mL, SantaCruz, USA), anti-CSE (20 ng/mL, SantaCruz, USA), anti-MPST (200 ng/mL, SantaCruz, USA) or anti-β-actin (20 ng/mL, Abcam, UK) (all in 1% BSA/TBS-T) overnight at 4 °C. The WB was then incubated with horse radish peroxidase (HRP)-anti-mouse/rabbit secondary antibody (1:2000 in 1% BSA/TBS-T, Sigma, UK) for 2 hr at room temperature. Immunodetection was visualized through enhanced chemiluminescence (Biorad, UK).

#### Confocal microscopy

To assess AgNP colocalization with and expression of H_2_S-synthezising enzymes (CBS, CSE and MPST) in N9 microglia cells following AgNPs treatment, cells seeded on cover slips in 24 well-plates were washed with freshly prepared Dulbecco’s phosphate buffer saline (PBS) at the experimental end-point, followed by fixation using ice cold methanol (x5 mins). The cells were rinsed with PBS, and blocked (1% BSA in PBS, pH 7.4) for 30 min at room temperature before they were incubated with primary antibodies (anti-CBS, -CSE or –MPST, 1:200 dilution) in blocking solution at 4 °C overnight. Following rinsing with PBS, cells were incubated with fluorescently-labelled secondary antibodies diluted in blocking solution (1:200 dilution) for 1 hr at room temperature. Following further rinsing, the nuclei were counter stained with DAPI. The glass cover slips were mounted onto microscope slides with SlowFade^®^ antifade reagent (Life Technologies, UK) and visualized using Leica SP5 inverted confocal microscope (Leica, Germany). As AgNPs have the capacity to reflect light, their uptake by cells can be monitored directly (*i*.*e*. without the use of fluorescent labelling) using confocal reflectance microscopy.

To quantify enzyme immunoreactivity, the fluorescent intensity of the enzyme in the cells was measured using Fiji (Image J) analysis software, and the data expressed as mean fluorescent intensity (MFI) ± standard deviation (SD). Forty individual cells were observed per sample, three independent samples were quantified per protein.

### Statistical analysis

The data were analysed by one-way ANOVA with Tukey’s *post-hoc* test or student’s t-test using SPSS (IBM) and GraphPad Prism 5 software. Differences between means were considered statistically significant at *p* < 0.05. Data are presented as mean ± standard error of the mean (SEM) of at least three independent experiments, unless otherwise stated.

## Additional Information

**How to cite this article**: Gonzalez-Carter, D. A. *et al*. Silver nanoparticles reduce brain inflammation and related neurotoxicity through induction of H_2_S-synthesizing enzymes. *Sci. Rep.* **7**, 42871; doi: 10.1038/srep42871 (2017).

**Publisher's note:** Springer Nature remains neutral with regard to jurisdictional claims in published maps and institutional affiliations.

## Supplementary Material

Supplementary Methods and Results

## Figures and Tables

**Table 1:** Summary of the physicochemical characteristics of AgNPs in DI water.

Average Size (TEM)	49.7 ± 10.5 nm in diameter
Capping Agent	Citrate
Surface Charge	−27.8 ± 0.1 mV

**Table 2:** Hydrodynamic diameter and zeta potential of AgNPs in water and cell culture media.

Solvent	Duration (h)	Average diameter (DLS), nm	Zeta potential, mV
Water	0	61.5	−27.8 ± 0.1
RPMI	1	379.9	−23.0 ± 0.8
DMEM	24	853.0	−18.5 ± 1.2

**Table 3:** Quantification of AgNP internalization into N9 microglia cells by ICP-AES following a 1 hr pulse/24 hr chase treatment (50 μg/mL).

Silver internalization (% of initial dose ± SEM)	29.96 ± 0.77
Silver internalization per cell (pg ± SEM)	30.52 ± 0.79
