# Supplementary material for: Silver nanoparticles reduce brain inflammation and related neurotoxicity through induction of H2S-synthesizing enzymes
Source: Sci Rep. 2017 Mar 2;7:42871. doi: 10.1038/srep42871 (PMC5333087; doi:10.1038/srep42871)
Supplement: Supplementary Methods and Results [file srep42871-s1.pdf]

Silver nanoparticles reduce brain inflammation and related neurotoxicity through induction of H_2S-synthesizing enzymes

Daniel A. Gonzalez-Carter[1, 2 a*], Bey Fen Leo[1, 3 a], Pakatip Ruenraroengsak[1, 4], Shu Chen[1], Angela E Goode[1], Ioannis G. Theodorou[1], Fan Chung[4], Raffa Carzaniga[5], Milo S.P. Shaffer[1, 6], David T. Dexter[2], Mary P. Ryan[1], Alexandra E. Porter[1*]

[1]Department of Materials and London Centre for Nanotechnology, Imperial College London, Exhibition Road, London, UK, SW7 2AZ

[2]Parkinson's Disease Research Unit, Centre for Neuroinflammation and Neurodegeneration, Division of Brain Sciences, Imperial College London, Hammersmith Hospital Campus, London, UK, W12 0NN

[3]Nanotechnology & Catalysis Research Centre (NANOCAT), University of Malaya, Kuala Lumpur 50603, Malaysia

[4]National Heart and Lung Institute, Imperial College London, Guy Scadding Building, Cale Street, London, UK, SW3 6LY

[5]Electron Microscopy Unit, The Francis Crick Institute, Lincoln's Inn Fields Laboratory, 44 Lincoln's Inn Fields, London, UK, WC2A 3LY

[6]Department of Chemistry and London Centre for Nanotechnology, Imperial College London, Exhibition Road, London, UK, SW7 2AZ

[a]These authors contributed equally to this work.
[*]Corresponding authors: daniel.gonzalez-carter08@imperial.ac.uk; a.porter@imperial.ac.uk

Supporting Methods

Commercially acquired silver nanoparticles: Citrate-capped silver nanoparticles (AgNP) were purchased from nanoComposix (California, USA). According to the manufacturer's specifications, AgNP in aqueous solution had a mean diameter of 51 $\pm$ 6 nm, as assessed by TEM, and a ζ-potential of -48 mV. The purity of the nanoparticles was 99.99% silver.

Supporting figures

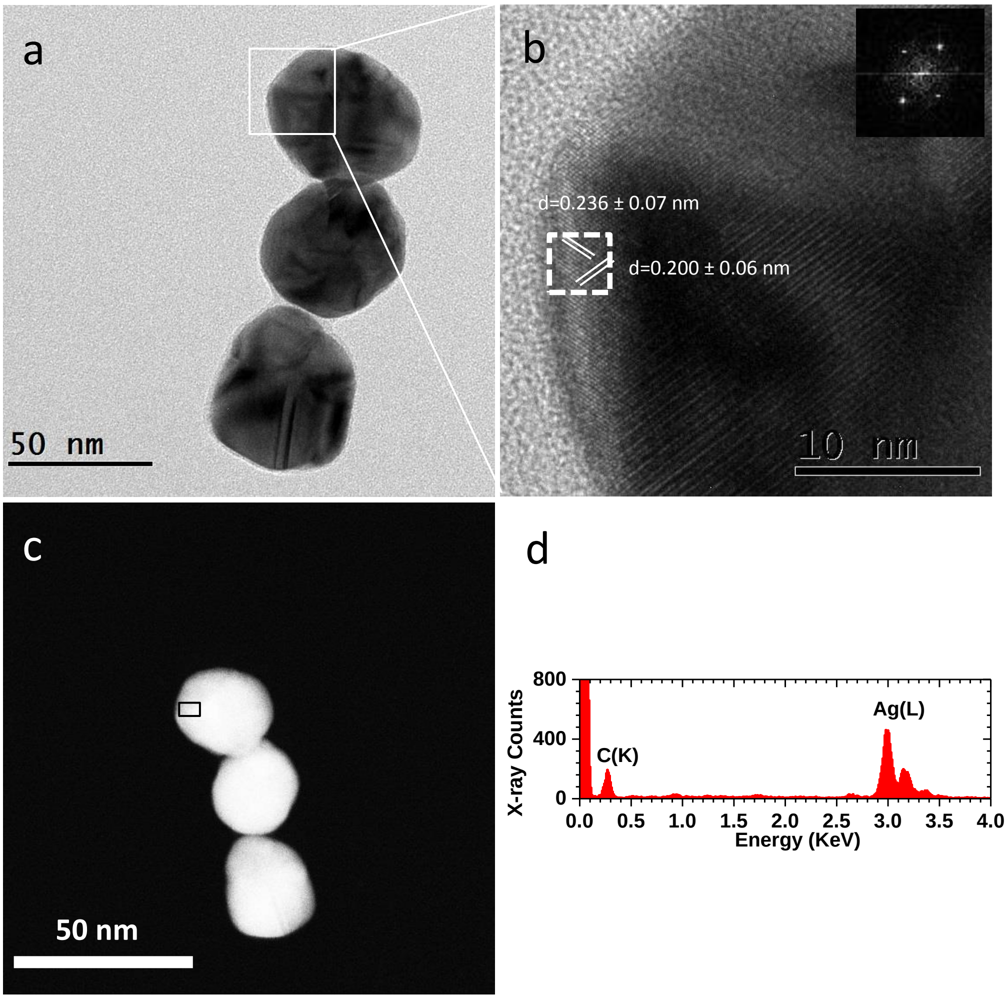

Fig. S1. TEM characterization of AgNPs incubated in RPMI cell culture media for 24 h at 37 °C. (a) Low resolution TEM image of the AgNPs. (b) HR-TEM analysis showing the lattice spacing of ~0.236 and ~0.200 nm, corresponding to interplanar spacing of bulk Ag (111) and (200) lattice planes, respectively (ref. #01-087-0597); inset is the FFT pattern taken from the dashed boxed area. (c) HAADF-STEM image taken from the same area as (a). (d) STEM-EDX spectrum collected from the boxed area in (c). TEM analyses were performed in a Jeol 2100F scanning/transmission electron microscope (S/TEM) operated at 200 kV, fitted with a X-MaxN Silicon Drift Detector with a detector sizes of 80 mm^2

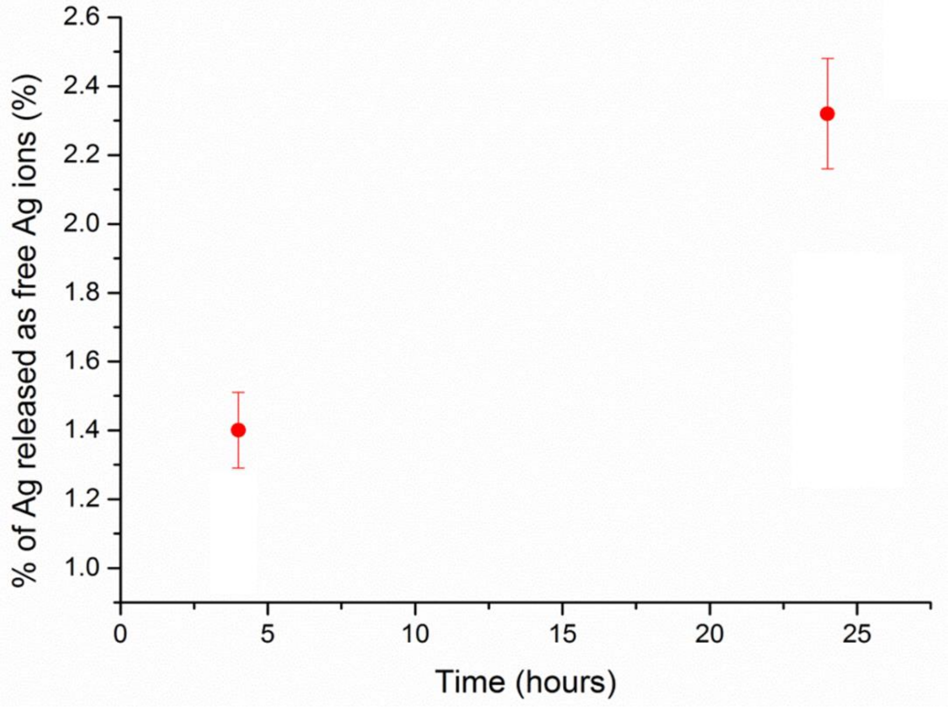

Fig. S2. Dissolution kinetics of AgNP. ICP-AES was employed to quantify the amount of free Ag^+ ions released from AgNPs in non-interacting perchlorate buffer solution (pH 5) after a 4h or 24h incubation. Results are displayed as mean $\pm$ SEM of three independent experiments.

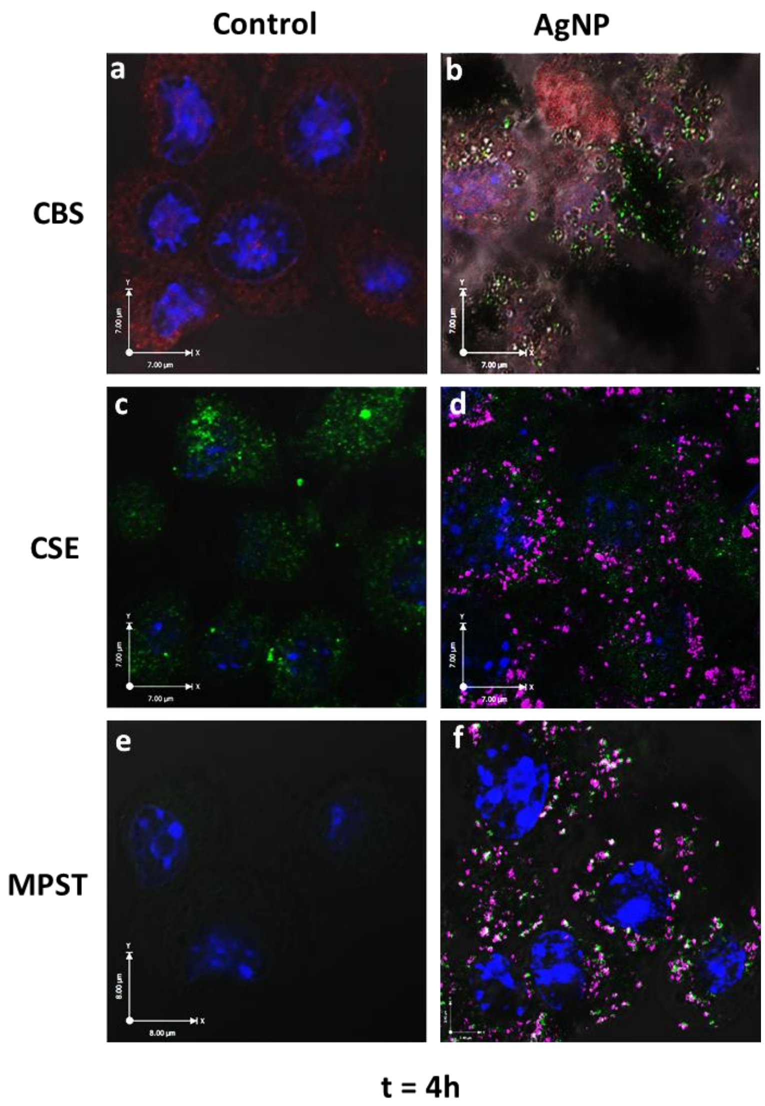

Fig. S3. The cellular uptake and distribution of AgNPs (50 µg/mL) inside N9 microglial cells combined with CBS, CSE and MPST enzyme expression following a 1h pulse treatment with a 4h chase period. Confocal images of CBS (a,b), CSE (c,d) and MPST (e,f) enzyme expression in control (a,c,e) and AgNP-treated (b,d,f) microglial cells. For images (a) and (b), CBS = red; AgNP = green; DAPI = blue. For images (c-f) CSE/MPST enzyme = green, AgNP = magenta, DAPI = blue. Images are representative of three independent experiments.

a)

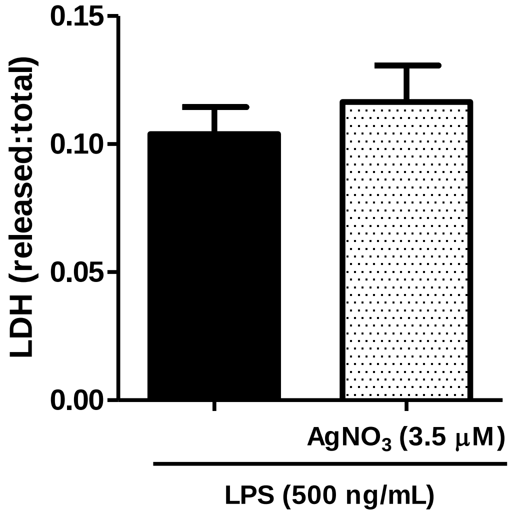

b)

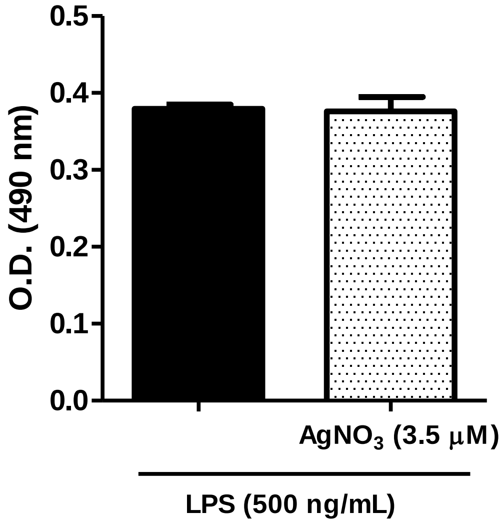

Fig. S4. Cytotoxicity of AgNO3 treatment on microglia cells. LDH release assay (a) and MTS metabolic activity assay (b) of N9 microglia treated with LPS (500 ng/mL) with or without $AgNO_3$ (3.5 μM) for 1hr (pulse) followed by a 24 hr chase period. Results are displayed as mean $\pm$ SEM of three independent experiments. Statistical significance was examined by a student's T-test with a significance value $p < 0.05$.

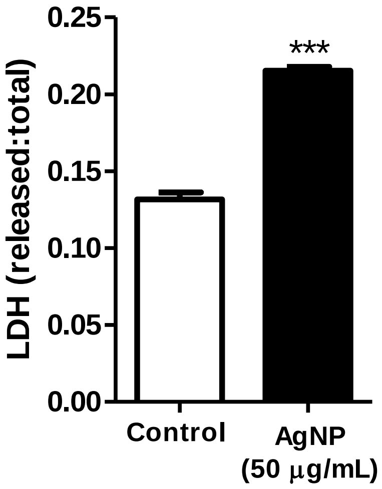

Fig. S5. Cytotoxicity of AgNP treatment on dopaminergic neuronal cells. N27 neurons were treated with AgNP (50 μg/mL) for 1 hr (pulse) followed by a 24 hr chase period, after which time-point cytotoxicity was assessed through an LDH release assay. Results are displayed as mean ± SEM of four independent experiments. *** denote $p < 0.005$ *vs.* control, as determined by a student's T-test.

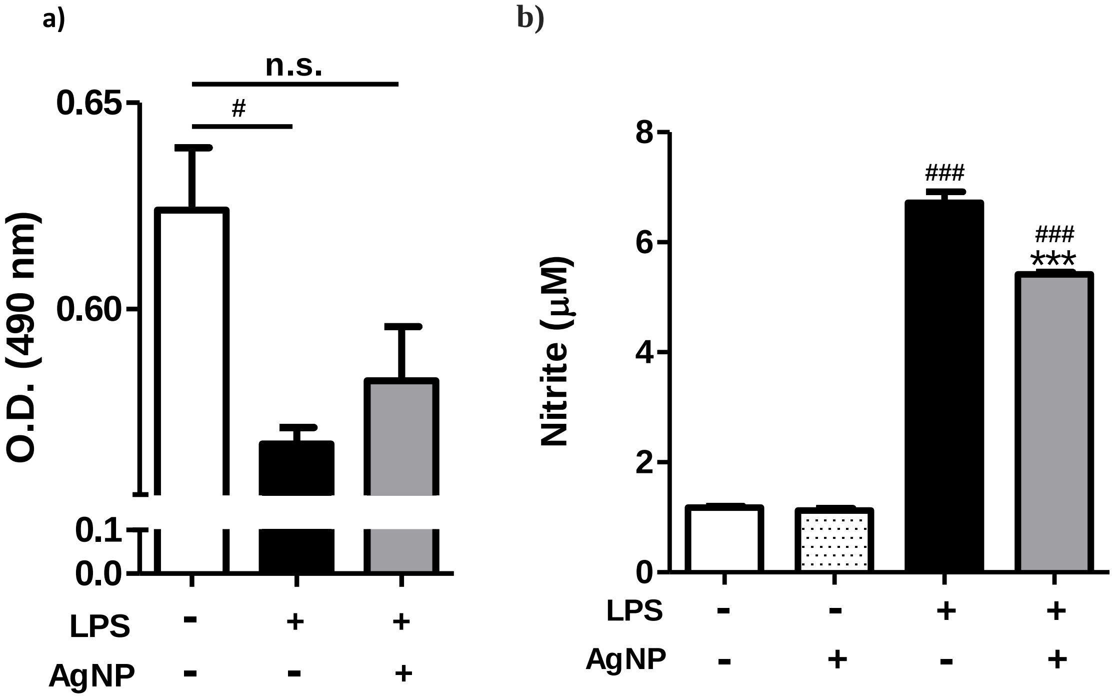

Fig. S6. Effect of commercially acquired AgNP on microglial neurotoxicity and inflammation. (a) Dopaminergic N27 neurons were incubated (48hr) with medium derived from control, LPS (500 ng/mL)- or LPS plus AgNP (50 μg/mL)-treated (24hr LPS treatment, 1hr pulse/24hr chase AgNP treatment) N9 microglia and neurotoxicity assessed through quantification of metabolic activity through an MTS assay. (b) Inflammation of control, LPS- or LPS plus AgNP-treated (1hr pulse/24hr chase) N9 microglia was assessed by quantification of nitrite production through a Griess assay. Results are displayed as mean ± SEM of three independent experiments. #, ### denote $p < 0.05$, 0.001 *vs.* control, respectively. *** denotes $p < 0.001$ *vs.* LPS treatment. Statistical significance was determined by a one-way ANOVA with a Tukey's *post-hoc* test.

Table S1: The lattice planes at the surface or the core of AgNPs/AgNWs (boxed areas in Fig, 2d) were identified by measuring the interplanar spacing from FFT.

Samples	Interplanar spacing from FFT (nm)	lattice plane
**c(i)**	2.093	Ag (200)
	2.402	Ag (111)
	2.328	Ag (111)
**c(ii)**	2.454	Ag_2S (112)
	2.779	Ag_2S (-112)
**c(iii)**	2.429	Ag_2S (112)
	2.085	Ag_2S (200)
**f(i)**	2.356	Ag_2S (-103)
	3.072	Ag_2S (111)
	2.796	Ag_2S (-112)
**f(ii)**	3.115	Ag_2S (111)
	2.594	Ag_2S(-121)
**f(iii)**	2.478	Ag_2S(112)
	2.574	Ag_2S (022)

Table S2: The crystal structure of silver and various silver compounds based on the Inorganic Crystal Structure Database.

Ag Crystal System (Cubic) - ICSD ref: 01-087-0597					
**h**	**k**	**l**	**d(Å)**	**2Theta (°)**	**I (%)**
1	1	1	2.359	38.115	100.0
2	0	0	2.043	44.299	45.7
2	2	0	1.445	64.443	22.5
3	1	1	1.232	77.397	22.2

AgCl crystal system (Cubic) - ICSD ref: 00-031-1238					
**h**	**k**	**l**	**d(Å)**	**2Theta (°)**	**I (%)**
2	0	0	2.774	32.244	100.0
2	2	0	1.962	46.234	50.0
1	1	1	3.203	27.831	50.0

Ag_2O crystal system (Cubic) - ICSD ref: 00-041-1104					
**h**	**k**	**l**	**d(Å)**	**2Theta (°)**	**I (%)**
1	1	1	2.729	32.791	100.0
2	0	0	2.360	38.067	28.0

Ag_2S crystal system (Monoclinic) - ICSD ref: 00-014-0072					
**h**	**k**	**l**	**d(Å)**	**2Theta (°)**	**I (%)**
-1	2	1	2.606	34.385	100.0
1	2	1	2.440	36.806	80.0
-1	0	3	2.383	37.719	75.0
-1	1	2	2.836	31.521	70.0
0	2	2	2.583	34.701	70.0
1	1	2	2.456	36.557	70.0
1	1	1	3.080	28.967	60.0
0	1	3	2.421	37.105	60.0
0	3	1	2.213	40.740	45.0
2	0	0	2.083	43.407	45.0
-1	1	1	3.437	25.902	35.0
-2	1	3	1.718	53.278	20.0
0	1	2	3.383	26.323	20.0

Table S3: List of ligands which are found in the CBS enzyme structures (source: European Protein Data Bank)

Cystathionine-β-synthase (CBS) (Enzyme Commission number, EC 4.2.1.22)

LIGAND	FORMULA	SYSTEMATIC NAME
PLP	$C_8\ H_{10}\ N\ O_6\ P$	PYRIDOXAL-5'-PHOSPHATE
HEM	$C_{34}\ H_{32}\ Fe\ N_4\ O_4$	PROTOPORPHYRIN IX CONTAINING FE
NA	Na	SODIUM ION
MPD	$C_6\ H_{14}\ O_2$	(4S)-2-METHYL-2, 4-PENTANEDIOL
ACT	$C_2\ H_3\ O_2$	ACETATE ION
PE4	$C_{16}\ H_{34}\ O_8$	2-(2-[2-(2-(2-[2-(2-ETHOXY-ETHOXY)-ETHOXY]-ETHOXY-ETHOXY)-ETHOXY]-ETHOXY)-ETHANOL
KOU	$C_{11}\ H_{15}\ N_2\ O_8\ P$	(E)-N-((3-hydroxy-2-methyl-5-[(phosphonooxy)methyl]pyridin-4-yl)methylidene)-L-serine
SEP	$C_3\ H_8\ N\ O_6\ P$	PHOSPHOSERINE
P1T	$C_{11}\ H_{15}\ N_2\ O_7\ P$	2-[((3-HYDROXY-2-METHYL-5-[(PHOSPHONOOXY)METHYL]PYRIDIN-4-YL)METHYL)AMINO]ACRYLIC ACID
EDO	$C_2\ H_6\ O_2$	1,2-ETHANEDIOL
OAS	$C_5\ H_9\ N\ O_4$	O-ACETYLSERINE

Table S5: List of ligands which are found in the CSE enzyme structures (source: European Protein Data Bank).

Cystathionine ϒ-lyase (CSE) (EC 4.4.1.1)

LIGAND	FORMULA	SYSTEMATIC_NAME
GOL	$C_3\ H_8\ O_3$	GLYCEROL
PLP	$C_8\ H_{10}\ N\ O_6\ P$	PYRIDOXAL-5'-PHOSPHATE
0JO	$C_{11}\ H_{13}\ N_2\ O_7\ P$	2-{[(E)-{3-hydroxy-2-methyl-5-[(phosphonooxy)methyl]pyridin-4-yl}methylidene]amino}prop-

		2-enoic acid
SO4	O_4 S	SULFATE ION
SER	C_3 H_7 N O_3	SERINE
PYR	C_3 H_4 O_3	PYRUVIC ACID
NAK	C_3 H_5 N O_2	AMINO-ACRYLATE
KOU	C_{11} H_{15} N_2 O_8 P	(E)-N-({3-hydroxy-2-methyl-5-[(phosphonooxy)methyl]pyridin-4-yl}methylidene)-L-serine
2AG	C_5 H_9 N O_2	(2S)-2-aminopent-4-enoic acid
NO3	N O_3	NITRATE ION
PEG	C_4 H_{10} O_3	DI(HYDROXYETHYL)ETHER
BCT	C H O_3	BICARBONATE ION
BME	C_2 H_6 O S	BETA-MERCAPTOETHANOL
CO3	C O_3	CARBONATE ION

Table S6: List of ligands which are found in the MPST enzyme structures (source: European Protein Data Bank).

3-mercaptopyruvate sulfurtransferase (MPST**)** (EC 2.8.2.1)

LIGAND	FORMULA	SYSTEMATIC_NAME
GOL	C3 H8 O3	GLYCEROL
SO4	O_4 S	SULFATE ION
PYR	C_3 H_4 O_3	PYRUVIC ACID
